# Synthesis, Crystal Structures, and Antimicrobial and Antitumor Studies of Two Zinc(II) Complexes with Pyridine Thiazole Derivatives

**DOI:** 10.1155/2020/8852470

**Published:** 2020-09-15

**Authors:** Zou Xun-Zhong, Feng An-Sheng, Zeng Fu-Ran, Lai Min-Cheng, Liao Yan-Zhi, Mei Meng, Li Yu

**Affiliations:** ^1^Guangdong Research Center for Special Functional Building Materials and Its Green Preparation Technology, Foshan Research Center for Special Functional Building Materials and Its Green Preparation Technology, Guangdong Industry Polytechnic, Guangzhou 510006, China; ^2^College of Light Industry and Food, Zhongkai University of Agriculture and Engineering, Guangzhou 510006, China; ^3^Wuhan Children's Hospital, Tongji Medical College, Huazhong University of Science and Technology, Wuhan 430015, China

## Abstract

Two pyridine thiazole derivatives, namely, 4-(pyridin-2-yl)-2-(2-(pyridin-2-ylmethylene)hydrazinyl)thiazole (L1) and 4-(pyridin-3-yl)-2-(2-(pyridin-4-ylmethylene)hydrazinyl)thiazole (L2), were afforded by a cyclization reaction between *α*-haloketone and thioamide, and their Zn(II) complexes were prepared by the reaction of ligands and corresponding metal salts, respectively, and characterized by X-ray diffraction and elemental analysis. Both crystals were obtained by ether diffusion and crystallized in a monoclinic system. The in vitro antimicrobial activity of the Zn(II) complexes and ligands was screened using the microplate reader method, and in vitro antitumor activities of the complexes were evaluated by MTT, with a view to developing new improved bioactive materials with novel properties. The biological activity studies of the compounds showed that the metal complexes were more active than the free ligands, and some compounds had absolute specificity for certain bacteria or cancer cell lines.

## 1. Introduction

**Scheme 1:**
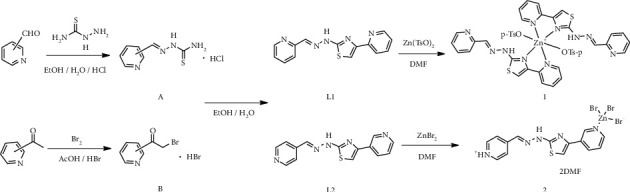
Synthesis route of ligands and complexes 1-2.

Thiazole ring and pyridine ring are structural units with extensive biological activities, such as antibacterial [1], antitumor [2], antiviral [3], anti-inflammatory [4], and hypoglycemic [5] activities. Hydrazone compounds have also attracted much attention in various fields, especially in pesticide and pharmaceutical fields due to their unique physiological activity and strong coordination ability [6, 7]. Therefore, the introduction of pyridine hydrazone group into molecule containing thiazole is likely to produce stronger biological activity according to the synergistic effect of drug combination principles. Because of the particularity of the structures, thiazole ligands containing pyridine and hydrazone groups with multiple coordination sites often have good metal coordination ability which can form stable complexes with different transition metal [8, 9]. Besides, the spatial conformation of the complex is determined by its coordinated modes, which have great influence on the biological properties of the compound [10–12]; the studies on transitional metal compounds of pyridine thiazole ligands have been of great significance. Co(III) complexes based on (1,3-selenazol-2-yl)- and (1,3-thiazol-2-yl)hydrazone were reported to have potent antimicrobial and antioxidant activity [13]. To further study the biological, physical, and chemical activities of these compounds and their Zn(II) complexes, two thiazole derivatives, L1 and L2, were synthesized by the cyclization reaction of pyridyl thiosemicarbazide and α-bromopyridine (Scheme 1). Complexes [Zn(**L1**)_2_(TsO)_2_] (1) and [Zn(H**L2**)Br_3_]·2DMF (2) were obtained by coordinating the ligands with Zn(TsO)_2_ and ZnBr_2_, respectively. The ligand L2 and complexes were characterized by elemental analysis (EA) and single-crystal diffraction. The inhibitory activities of these compounds against seven pathogenic bacterial strains, namely, *Escherichia coli* (*E. coli*, ATCC 25922), *Staphylococcus aureus* (*S. aureus*, CMCC(B) 26003), *Salmonella typhimurium* (*S. typhimurium*, CMCC(B) 50071), *Bacillus subtilis* (*B. subtilis*, ATCC 6633), *Shigella flexneri* (*Sh. flexneri*, CMCC(B) 51572), *Vibrio parahaemolyticus* (*V. parahaemolyticus*, ATCC 17802), and *Pseudomonas aeruginosa* (*P. aeruginosa*, ATCC 9027), and four human cancer cell lines (human lung cancer cells A549, human breast cancer cells BT-20, human ovarian cells MCF-7, and human osteosarcoma cells U20S), were tested.

## 2. Experimental Setup

### 2.1. Materials and Methods

All the starting materials and reagents in this work were obtained commercially and used without further purification. Elemental analyses were performed on a vario EL analyzer (Elementar, Germany) and IR spectra on an Avatar 330 FT-IR spectrometer (Thermo Nicolet) with potassium bromide pellets. ^1^H NMR spectra were determined by Bruker AVANCE-III 500 at 300–400 MHz, and MestReNova software was used for data analysis. Chemical shifts (*δ* values) and coupling constants (*J* values) are reported as ppm and Hz, respectively. The following abbreviations are used: s, singlet; d, doublet; t, triplet; q, quartet; m, multiplet; br, broad. Antimicrobial tests were performed on a SpectraMax® ABS Absorbance Reader (Molecular Devices). Single-crystal X-ray diffraction was carried out by a Bruker Smart Apex-CCD and Agilent SuperNova diffractometer. The UV-Vis spectra were recorded on an Agilent Cary 60 spectrophotometer.

### 2.2. Synthesis of Ligands L1 and L2

A mixture of 2-pyridine formaldehyde (10.7 g, 0.1 mol), 50 mL ethanol, 50 mL H_2_O, and 5 mL 36% concentrated hydrochloric acid was added to a 250 mL three-neck flask with magnetic agitator and reflex tube at 25°C. Thiosemicarbazide (10.9 g, 0.12 mol) in 10 mL ethanol was added slowly, and the solution immediately became yellow solid. After being stirred for 0.5 h and refluxed for 2 h, the solid was filtered and rinsed with ethanol to give the N-2-pyridine-methyl-thiosemicarbazide hydrochloride (20.2 g, yield 93.5%, **Compound A**) as a yellow acicular crystal [14].

2-Acetyl pyridine (12.1 g, 0.1 mol) was added to a solution of ice acetic acid (85 mL) and hydrobromic acid (40%, 13 mL), and the mixture was stirred for 10 min under ice bath. After being completely cooled, bromide (0.1 mol, 5.2 mL) was added three times slowly. Then, the reaction mixture was stirred for 0.5 h at 0°C, 2 h at 40°C, and 2 h at 75°C, and the solution gradually changed from dark brown to light yellow with white crystal solid precipitation. After the reaction system was cooled to room temperature, 80 mL ethyl ether solution was added. Then the mixture was stirred continuously for 10 min, filtered, and washed with dry ethyl ether three times to give bromo-2-acetylpyridine hydrobromide (19.5 g, yield 90.3%, **Compound B**) as a white crystal [15].

**N-Pyridin-2-ylmethylene-N'-(4-pyridin-2-yl-thiazol-2-yl)-hydrazine (L1):** N-2-pyridine methylene-thiosemicarbazide hydrochloride (10.8 g, 0.05 mol) and acetyl pyridine hydrobromide (10.8 g, 0.05 mol) were added to 160 mL 50% ethanol solution at 25°C. After being refluxed for 2 h, the mixture was cooled to room temperature and adjusted to neutral with 10% sodium hydroxide solution. The resultant solid was filtered, washed repeatedly with distilled water, and recrystallized with methanol to give N-pyridin-2-ylmethylene-N′-(4-pyridin-2-yl-thiazol-2-yl)-hydrazine (9.2 g, yield 65.7%) as a yellow crystalline solid. IR (KBr, cm^−1^): 3139.99(m), 3053.18(m), 2970.57(m), 2857.63(m), 2770.29(w), 1588.44(s), 1570.92(d), 1514.39(m), 1475.46(s), 1435.06(s), 1422.61(m), 1353.43(m), 1303.55(m), 1275.00(m), 1263.80(m), 1148.39(s) cm^−1^. ^1^H NMR (400 MHz, DMSO-d6) *δ* 12.45 (s, 1H), 8.59 (dd, *J* = 3.6, 2.3 Hz, 2H), 8.08 (s, 1H), 7.93-7.81 (m, 4H), 7.59 (s, 1H), 7.41-7.25 (m, 2H). ESI-MS *m*/*z*(%): 281.8 (M^+^+1). Anal. Calcd. For C_14_H_11_N_5_S: C, 59.77; H, 3.94; N, 24.89; S, 11.40. Found: C, 59.69; H, 3.98; N, 24.92; S, 11.36. UV-Vis (DMSO, *λ*_max_, nm): 255.0, 340.0.

**4-(Pyridin-3-yl)-2-(2-(pyridin-4-ylmethylene)hydrazinyl)thiazole (L2) was prepared in a similar way to ligand L1,** with 3-pyridine formaldehyde, 3-acetyl pyridine instead of 2-pyridine formaldehyde, 2-acetyl pyridine. **L2**, yellow power, yield 12.2 g (86.5%). IR (KBr, cm^−1^): 3200.00(m), 3079.41(m), 2743.00(w), 1636.84(m), 1599.83(s), 1561.72(s), 1499.00(s), 1462.66(s), 1407.26(m), 1375.20(m), 1283.75(m), 1212.35(m), 1186.00(m), 1169.97(m), 1123.58(m) cm^−1^. ^1^H NMR (400 MHz, DMSO-d6) *δ* 13.07 (s, 1H), 9.14 (d, *J* = 1.6 Hz, 1H), 8.77 (d, *J* = 6.5 Hz, 2H), 8.61 (dd, *J* = 5.0, 1.5 Hz, 1H), 8.38 (d, *J* = 8.1 Hz, 1H), 8.13 (s, 1H), 7.97 (d, *J* = 6.5 Hz, 1H), 7.76 (s, 1H), 7.62 (dd, *J* = 8.0, 5.0 Hz, 1H). ESI-MS *m*/*z*(%): 281.8 (M^+^+1). Anal. Calcd. For C_14_H_11_N_5_S: C, 59.77; H, 3.94; N, 24.89; S, 11.40. Found: C, 59.73; H, 3.97; N, 24.90; S, 11.36. UV-Vis (DMSO, *λ*_max_, nm): 255.0, 365.0.

### 2.3. Synthesis of Complexes 1-2

**Complex 1.** To a solution of 4-(pyridin-2-yl)-2-(2-(pyridin-2-ylmethylene)hydrazinyl)thiazole (**L1**) (28 mg, 0.1 mmol) in DMF (2 mL), Zn(TsO)_2_ (40.7 mg, 0.1 mmol) in DMF (2 mL) was added slowly dropwise. The solution was filtered, and yellowish crystal (42.2 mg) was obtained in a few days by the diffusion of diethyl ether in a wild-mouth bottle. The yield was 41.2%. IR (KBr, cm^−1^): 3216.90(m), 3071.80(m), 1605.36(s), 1592.50(s), 1574.47(d), 1494.73(m), 1470.91(s), 1445.51(m), 1432.01(m), 1376.73(m), 1311.65(m), 1284.21(m), 1155.66(s), 1107.48(s), 1029.73(m), 1005.69(s). ^1^H NMR (400 MHz, DMSO-d6) *δ* 12.41 (s, 1H), 8.60 (s, 2H), 8.13 (s, 2H), 7.90 (s, 4H), 7.48 (d, *J* = 8.1 Hz, 2H), 7.39 (s, 2H), 7.11 (d, *J* = 7.8 Hz, 2H), 2.29 (s, 3H). Anal. Calcd. For C_42_H_36_N_10_O_6_S_4_Zn: C, 51.98; H, 3.74; N, 14.43; S, 13.22. Found: C, 51.82; H, 3.72; N, 14.39; S, 13.18. UV-Vis (DMSO, *λ*_max_, nm): 255.0, 335.0.

**Complex 2.** To a solution of 4-(pyridin-3-yl)-2-(2-(pyridin-4-ylmethylene)hydrazinyl)thiazole (**L2**) (28 mg, 0.1 mmol) in CH_3_OH (5 mL), ZnBr_2_ (22.5 mg, 0.1 mmol) in CH_3_OH (5 mL) was added. The precipitate was filtered, washed with CH_3_OH three times, and redissolved with DMF (5 mL). By the diffusion of diethyl ether, the yellowish crystal (20.8 mg) was obtained in a few days. The yield was 44.8%. IR (KBr, cm^−1^): 3245.21(m), 3096.59(m), 1593.25(s), 1567.21(s), 1500.00(m), 1449.70(s), 1410.93(m), 1370.81(s), 1306.35 (m), 1288.54(s), 1246.09(d), 1192.70(m), 1161.98(s), 626.84(s). ^1^H NMR (400 MHz, DMSO-d6) *δ* 12.96 (s, 1H), 9.12 (s, 1H), 8.73 (d, *J* = 6.4 Hz, 1H), 8.58 (d, *J* = 6.3 Hz, 1H), 8.32 (d, *J* = 7.7 Hz, 1H), 8.10 (s, 1H), 7.89 (d, *J* = 6.3 Hz, 1H), 7.71 (s, 1H), 7.56 (dd, *J* = 8.3, 5.0 Hz, 1H). Anal. Calcd. For C_20_H_26_Br_3_N_7_O_2_SZn: C, 32.74; H, 3.57; N, 13.36; S, 4.37. Found: C, 32.79; H, 3.55; N, 13.27; S, 4.35. UV-Vis (DMSO, *λ*_max_, nm): 260.4, 370.0.

### 2.4. X-Ray Structure Determination

Crystals were selected appropriately and mounted on a glass fiber in a random orientation. A single crystal of ligand **L1** was mounted on a Bruker Smart Apex-CCD diffractometer with CuK*α* radiation (*λ* = 1.54178 Å) at 293 K, while the other crystals were carried out on an Agilent SuperNova diffractometer with CuK*α* radiation (*λ* = 1.54178 Å) and with MoK*α* radiation (*λ* = 0.71073 Å) at 100K, respectively. The crystallographic data and structure refinement summary of ligands **L1**, **L2** and **complexes 1-2** are listed in Table 1. Using OLEX2 [16], the structure was solved by direct methods using the SHELXS program [17] and refined by full-matrix least-squares techniques SHELXL-2014 [18] on *F*^2^. All nonhydrogen atoms were refined anisotropically. DIAMOND was used for molecular graphics [19].

### 2.5. Antimicrobial Activity Studies

Qualitative analysis for screening of antibacterial activity was carried out by the microplate reader method [20–22]. Mueller–Hinton agar plates were seeded with indicator bacteria, *Escherichia coli* (*E. coli*, ATCC 25922), *Staphylococcus aureus* (*S. aureus*, CMCC(B) 26003), *Salmonella typhimurium* (*S. typhimurium*, CMCC(B) 50071), *Bacillus subtilis* (*B. subtilis*, ATCC 6633), *Shigella flexneri* (*Sh. flexneri*, CMCC(B) 51572), *Vibrio parahaemolyticus* (*V. parahaemolyticus*, ATCC 17802), and *Pseudomonas aeruginosa* (*P. aeruginosa*, ATCC 9027), and cultured for 24 h at 37°C. Plates were inoculated with bacteria adjusted to 0.5 McFarland turbidity standards (10^8^ cfu/mL). All compounds were dissolved in DMSO (100 *μ*g/mL as an initial concentration) and tested against the ten pathogenic bacteria strains for their inhibitory activity. The antimicrobial activity of the compounds was expressed as the bacteriostatic rate (%) and determined by the absorbance of the bacterial samples using an enzyme-labeled instrument. Subsequently, compounds with high inhibitory rate (+++) were selected for minimum inhibitory concentration (MIC) determination using double dilution method with concentrations ranging from 3.125 to 50 *μ*g/mL. The MIC was recorded as the lowest concentration at which inhibitory rate was greater than 95%. As a blank group, only DMSO was added to the wells, and Ciprofloxacin was used as positive control for antibacterial activity. All tests and analyses were run in duplicate, and the results obtained were averaged. The percentage inhibition was calculated using the following equation:

(1) $$\begin{array}{c} \text{inhibition}\left(\%\right)=\frac{Abs_{\text{blank}}-\left(Abs_{\text{sample}}-Abs_{\text{control}}\right)}{Abs_{\text{blank}}}\times 100, \end{array}$$

where *Abs*_*blank*_ is the absorbance of DMSO + blank medium, *Abs*_*control*_ is the absorbance of sample + blank medium, and *Abs*_*sample*_ is the absorbance of sample (test samples/standard) + bacterial suspension in medium.

### 2.6. Antitumor Activity Studies

The viability of the cell lines was tested using 3-(4,5-dimethylthiazol-2-yl)-2,5-diphenyltetrazolium bromide (MTT) assay [23, 24]. The compounds were dissolved in DMSO and diluted with water to the required concentration (160 *μ*g/mL, 80 *μ*g/mL, 40 *μ*g/mL, 20 *μ*g/mL, 10 *μ*g/mL, 5 *μ*g/mL, 0 *μ*g/mL). Human lung cancer cells (A549), human breast cancer cells (BT-20), human ovarian cell cells (MCF-7), and human osteosarcoma cells (U20S) cells were kindly provided by Stem Cell Bank, Chinese Academy of Sciences, and were plated in 96-multiwell plates (104 cells/well) for 24 hours before treatment with different concentrations of complexes to allow attachment of cell to the wall of the plates. Then, the cells were cultured using MEM, DMEM, F-12K, and DMEM/F-12(GIBCO) culture medium containing 10% fetal bovine serum in a 5% (volume fraction) CO_2_, 37°C saturated humidity incubator for 48 hours, respectively. Cells were fixed, washed, and stained with MTT. Cell growth inhibition was determined by measuring the absorbance of each well at 570 nm with a Tecan Infinite M1000 Pro Microplate Reader (Männedorf, Switzerland). GraphPad Prism version 7.0 program was used for data processing, and IC50 was obtained by fitting the nonlinear regression model with S-shaped dose response in the program.

## 3. Results and Discussion

### 3.1. Solution Stability of Complexes 1-2

The essential prerequisite for the biological evaluation of metal complexes is their stability in solution. In the light of this fact, **complexes 1-2** were dissolved in DMSO and DMSO-d6 (a solvent used for the preparation of stock solutions for biological evaluation), and their UV-Vis and NMR spectra, respectively, were recorded immediately after dissolution, as well as after 48 h of standing in the dark at ambient temperature. From NMR measurements (Figure S1), it can be concluded that the corresponding N-pyridine and N-thiazole remained coordinated to the Zn(II) ion during 48 h and that DMSO coordination did not occur. In the UV-Vis spectra (Figure S3), the shape of spectra and position of the absorption maxima (*λ*_max_ = 255.0, 335.0 nm for **complex 1**; *λ*_max_ = 260.4, 370.0 nm for **complex 2**) remained unmodified. Taken together, obtained spectroscopic data indicated the sufficient stability of **complexes 1-2** in DMSO solution.

### 3.2. FTIR Spectral Elucidation

The infrared absorption peaks of the main functional groups on ligands **L1** and **L2** and complexes **1–3** are listed in Table 2. Additionally, as shown in Figure S2, the peak at 2970.57 cm^−1^ was the stretching vibration peak of the C-H bond in the N=C-H group, and the peaks at 2857.63 and 2770.29 cm^−1^ belonged to the stretching vibration peak of the C-H bond on the thiazole ring, while that at 1263.80 cm^−1^ was the stretching vibration peak of the Schiff base C=N bond. The peaks at 1148.39 cm^−1^ were attributed to the stretching vibration of the C-N bond. In compound **L2**, the peak at 2743.00 cm^−1^ belonged to the stretching vibration peak of the C-H bond on the thiazole ring, whereas that at 1283.75 cm^−1^ was the stretching vibration peak of the Schiff base C=N bond. The peaks at 1186.00, 1123.58, and 1169.97 cm^−1^ were attributed to the stretching vibration of the C-N bond. In complex **1**, the peaks at 1605.36, 1574.47, 1445.51, and 1494.73 cm^−1^ were caused by the skeleton vibration of the benzene ring. The peak at 1155.66–1005.69 cm^−1^ belonged to the antisymmetric stretching vibration and symmetric stretching vibration of the SO_3_ group in p-toluene sulfonate. In complex **2**, the stretching vibration peaks of the C-N bond were 1192.70 and 1161.98 cm^−1^, whereas 626.84 cm^−1^ might belong to the characteristic absorption peak of Zn-Br.

### 3.3. X-Ray Crystal Structure

**Figure 1:**
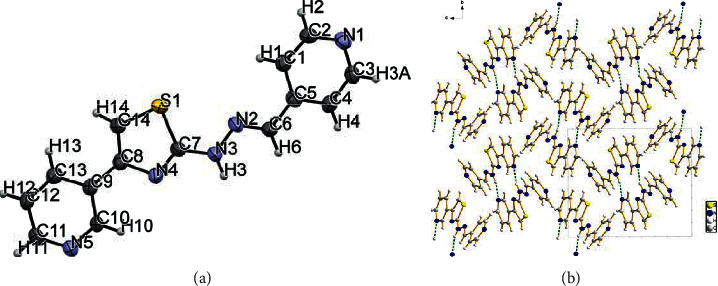
(a) The structure of ligand L2. (b) Molecular accumulation figure viewed along a-axis in L2 (green lines represent the H-bonds).

Suitable single crystals of ligand **L2** and complexes **1-2** for X-ray diffraction studies were obtained by the diffusion of diethyl ether into a DMF solution at room temperature. Ligand **L2** was crystallized in a monoclinic system, and the spatial group was P2_1_/*n*. The selected bond lengths and bond angles of **L2** are listed in Table 3. The N(3)–N(2) bond (1.360(2)Å) has the same length as the N(3)–C(7) bond. The molecular structure of L2 and its packing diagram are depicted in Figure 1. In the crystal of **L2**, the molecule is in the E-conformation, and the supramolecular arrangement is directed mainly by strong N(3)–H(3)···N(5) hydrogen bonds between two molecules (Table 4). The torsion angles of C(13)-C(9)-C(8)-N(4) (172.59°) and C(10)-C(9)-C(8)-C(14) (174.37°) indicate that the thiazole ring is almost coplanar with the neighboring pyridine ring.

**Figure 2:**
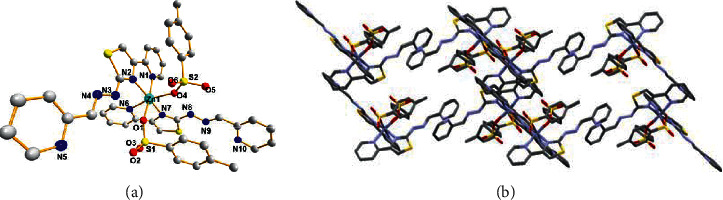
(a) View of the coordination environment of Zn(II) ions in 1. (b) Molecular accumulation figure viewed along b-axis in 1.

Complex **1** crystallized in a monoclinic unit cell, P2_1_/*c* space group. The selected bond lengths and bond angles of complex **1** are listed in Table 5. As shown in Figure 2, the Zn(II) atom occupies the center of inversion with a coordination model of ML_2_, and it is coordinated by four nitrogen atoms from two ligands, two oxygen atoms from two p-toluene sulfonic acid ions. The distances of Zn(1)–O(1) and Zn(1)–O(4) are 2.153(4) and 2.117(5), respectively. The distances of Zn(1)–N(1), Zn(1)–N(2), Zn(1)–N(6), and Zn(1)–N(7) are 2.140(6), 2.089(6), 2.175(5), and 2.085(5)Å, respectively. The S1 atom in thiazole ring was not coordinated with the metal ion. The N(2)–Zn(1)–O(4), N(2)–Zn(1)–N(6), and N(7)–Zn(1)–O(1) bond angles are all nearly 90°, indicating that the complexes have nearly perfect hexahedron geometry as expected. The bond distances and angle values of **2** are in good agreement with the related Zn(II) complexes.

**Figure 3:**
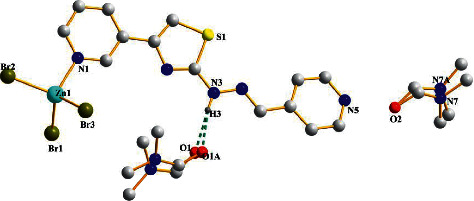
The structure of 2 (green lines represent the H-bonds).

The selected bond lengths and angles for complex **2** are summarized in Table 6. A deformed tetrahedron geometry around the central atom is confirmed by the Br(1)–Zn(1)–Br(2), Br(3)–Zn(1)–Br(2), Br(1)–Zn(1)–Br(3), and N(1)–Zn(1)–Br(1) bite angles, which differ a little as expected for atoms coordinated in cis-positions, and also by the Zn(1)–N1 bond (2.073(4)), which is slightly shorter than the Zn(1)–Br bonds (2.3834(8), 2.3878(9), 2.3715(9)Å, respectively) due to the smaller covalent radius of nitrogen. Nevertheless, the bond distances around the Zn(II) atom are similar to those observed in similar Zn(II) complexes as reported in the Crystallography Open Database [25]. Two disordered DMF molecules were not involved in the coordinate system with 35% and 45% occupancy factors, respectively (Figure 3, Table 7). Additionally, intramolecular hydrogen bonds N(3)–H(3)···O(1) and N(3)–H(3)···O(1A) were observed between ligand **L2** and one disordered DMF molecule with the distance of 2.793(16) and 2.740(3)Å in the crystal structure.

### 3.4. Biological Activity

The antibacterial activity of the ligands and complexes was presented as inhibition rate, and those with high inhibitory rate (+++) were selected for minimum inhibitory concentration (MIC) determination. As shown in Table 8, the complexes had a wider broad spectrum and stronger antibacterial property than the ligands, and such enhanced activity of metal chelates is due to faster diffusion of metal complexes as a whole through the cell membrane or due to combined activity effect of the metal and the ligand. To further measure the MIC, CIPRO was used as the positive control. Compared with the CIPRO, complex **2** had a better or the same inhibitory effect on *E. coli*, *Sh. flexneri*, and *P. aeruginosa* with the MIC of 3.13 *μ*g·mL^−1^, 3.13 *μ*g·mL^−1^, and 6.25 *μ*g·mL^−1^, respectively, while complex **1** held a stronger inhibitory effect on *S. typhimurium* and *B. subtilis* with the MIC of 6.25 *μ*g·mL^−1^ and 3.13 *μ*g·mL^−1^, respectively (Table 9). These results indicated that the complexes possess antibacterial activity inhibiting multiplication process of the microbes by blocking their active sites [26, 27].

The *in vitro* antitumor activity of the compounds against human lung cancer cells (A549), human breast cancer cells (BT-20), human ovarian cancer cells (MCF-7), and human osteosarcoma cells (U20S) was measured via MTT assay. In general, the complexes showed a stronger antitumor activity than the corresponding ligands (Table 10). Between the two complexes, complex **2** exerted a more effective antitumor activity with the lower IC_50_ value. Among the four cancer cell lines, complex **2** had the most significant effect on A549 with the IC_50_ value of 4.48 *μ*g/mL. The result indicated the enhancement of the antitumor activity upon coordination. The enhancement of antitumor activity may be attributed to the fact that the positive charge of the metal increased the acidity of coordinated ligand that bears protons, leading to stronger hydrogen bonds which enhanced the biological activity [28]. Despite the conformed antitumor activity, a detailed molecular mechanism against cancer cell lines of the ligands and the complexes remains to be further elucidated.

## 4. Conclusion

In this paper, We have reported the preparation, characterization, and biological activities of new thiazole-hydrazone derivatives based on pyridine and their two Zn(II) complexes. Single-crystal X-ray diffraction analysis indicated that both ligand **L2** and complexes **1-2** had mononuclear molecular structure. The in vitro antimicrobial activity of the complexes was screened using the microplate reader method, and in vitro antitumor activities of the complexes were evaluated by MTT. The results showed that the complexes coordinated with Zn^2+^ had a better antimicrobial activity and antitumor activity than the corresponding ligands due to the lipophilic nature of the metal ions in complexes, which might provide valuable information for further designing and synthesizing new antimicrobial and antitumor agents.

## Figures and Tables

**Table 1:** Crystallographic data and structure refinement summary for ligand L2 and complexes 1-2.

	L2	1	2
Empirical formula	C_14_H_11_N_5_S	C_42_H_36_N_10_O_6_S_4_Zn	C_20_H_26_Br_3_N_7_O_2_SZn
Mw	281.34	970.44	733.64
Crystal system	Monoclinic	Monoclinic	Monoclinic
*a* (Å)	8.5684(5)	17.614(2)	8.3543(5)
*b* (Å)	11.3512(6)	20.6571(13)	20.0129(13)
*c* (Å)	13.5596(8)	17.620(2)	16.2751(11)
*α* (°)	90	90	90
*β* (°)	107.149(6)	112.260(15)	94.611(6)
*γ* (°)	90	90	90
*V* (Å^3^)	1260.19(13)	5933.2(12)	2712.3(3)
Space group	P2_1_/*n*	P2_1_/*c*	P2_1_/*n*
Density (g·cm^−3^)	1.483	1.046	1.797
*Z*	4	4	4
*μ* (mm^−1^)	2.254	0.597	5.433
Temperature (K)	100	100	100
*F*(000)	584	1857	1448
Crystal size (mm^3^)	0.12 × 0.1 × 0.08	0.12 × 0.11 × 0.08	0.12 × 0.11 × 0.1
Radiation	(Cu-K*α*) 1.54184	(Mo-K*α*) 0.71073	(Mo-K*α*) 0.71073
Index ranges	−10 ≤ *h* ≤ 8, −13 ≤ *k* ≤ 9, −16 ≤ *l* ≤ 16	−24 ≤ *h* ≤ 22, −28 ≤ *k* ≤ 22, −23 ≤ *l* ≤ 24	−10 ≤ *h* ≤ 11, −25 ≤ *k* ≤ 26, −21 ≤ *l* ≤ 11
*θ* range for data collection (°)	5.1730–72.8960	2.2960–23.1730	2.7030–26.5730
Goodness-of-fit on *F*^2^	1.028	1.025	1.047
Reflections collected	4756	33034	14560
Independent reflections	2468 [*R*_int_ = 0.0399, *R*_sigma_ = 0.0544]	14148 [*R*_int_ = 0.1101, *R*_sigma_ = 0.2110]	6500 [*R*_int_ = 0.0455, *R*_sigma_ = 0.0794]
Final *R* indexes [I ≥ 2*σ*(*I*)] *R*_1_, *ωR*_2_	0.0430, 0.0985	0.1018, 0.2400	0.0571, 0.1240
Final *R* indexes [all data] *R*_1_, *ωR*_2_	0.0578, 0.1063	0.1860, 0.2872	0.0897, 0.1391
Data/restraints/parameters	2468/0/181	14148/85/498	6500/101/398
Largest diff. peak/hole (e Å^−3^)	0.685/−0.490	1.691/−2.571	0.86/−1.72

**Table 2:** Infrared absorption peaks of the main functional groups on ligands **L1** and **L2** and complexes **1–3**.

	L1	L2	1	2
Stretching vibration peak (N-H)	3139.99 cm^−1^	3200.00 cm^−1^	3216.90 cm^−1^	3245.21 cm^−1^
Skeleton vibration peak (pyridine)	1353.43, 1588.44, 1514.39, 1475.46 cm^−1^	1462.66, 1499.00, 1599.83, 1636.84 cm^−1^	1592.50, 1376.73 cm^−1^	1567.21, 1593.25, 1500.00, 1449.70 cm^−1^
Skeleton vibration peak (Thiazole)	1570.92, 1435.06, 1422.61 cm^−1^	1561.72, 1407.26, 1375.20 cm^−1^	1470.91, 1432.01, 1311.65 cm^−1^	1410.93, 1370.81, 1306.35 cm^−1^
Stretching vibration peak (C-H on pyridine)	3053.18 cm^−1^	3079.41 cm^−1^	3071.80 cm^−1^	3096.59 cm^−1^
Stretching vibration peak (C=N on ring)	1303.55, 1275.00 cm^−1^	1212.35 cm^−1^	1284.21 cm^−1^	1288.54, 1246.09 cm^−1^
In-plane oscillations peak (C-H on pyridine and thiazole ring)	1100–400 cm^−1^	1100–400 cm^−1^	1000–400 cm^−1^	1100–600 cm^−1^

**Table 3:** Selected bond lengths (Å) and bond angles (°) of L2.

Bond	Dist.
S(1)–C(7)	1.743(2)
S(1)–C(14)	1.724(2)
N(4)–C(8)	1.390(3)
N(3)–C(7)	1.360(3)
N(5)–C(10)	1.345(3)
N(5)–C(11)	1.336(3)
N(4)–C(7)	1.303(3)
N(2)–C(6)	1.279(3)
N(3)–N(2)	1.360(2)

Angle	(°)
C(14)–S(1)–C(7)	88.1(1)
C(7)–N(4)–C(8)	109.65(18)
N(2)–N(3)–H(3)	121.300
C(13)-C(9)-C(8)-N(4)	172.59
N(3)–C(7)–S(1)	120.47(15)
N(2)–C(6)–C(5)	120.4(2)
N(2)–C(6)–H(6)	119.800
(10)-C(9)-C(8)-C(14)	174.37
N(2)–N3—C7	117.43(18)
C(7)–N(3)–H(3)	121.300
C(6)–N(2)–N(3)	116.83(18)

**Table 4:** Hydrogen bonds for the L2 (Å and °).

D–H···A	D–H	H···A	D···A	D–H···A
N(3)–H(3)···N(5)	0.8800	2.0440	2.864	159.04

**Table 5:** Selected bond lengths (Å) and bond angles (°) of complex 1.

Bond	Dist.
Zn(1)–N(2)	2.089(6)
Zn(1)–O(4)	2.117(5)
Zn(1)–N(6)	2.175(5)
Zn(1)–O(1)	2.153(4)
Zn(1)–N(7)	2.085(5)
Zn(1)–N(1)	2.140(6)
S(1)–O(1)	1.487(4)
S(2)–O(4)	1.487(5)
N(9)–N(8)	1.368(7)

Angle	(°)
N(2)–Zn(1)–O(4)	90.2(2)
N(2)–Zn(1)–N(6)	97.4(2)
N(2)–Zn(1)–O(1)	94.40(19)
N(7)–Zn(1)–O(4)	95.46(19)
N(7)–Zn(1)–N(6)	78.01(18)
N(2)–Zn(1)–N(1)	78.0(2)
O(4)–Zn(1)–N(6)	91.01(18)
O(4)–Zn(1)–O(1)	87.19(16)
N(7)–Zn(1)–O(1)	90.43(17)
N(7)–Zn(1)–N(1)	96.8(2)
O(4)–Zn(1)–N(1)	166.75(18)
O(1)–Zn(1)–N(6)	168.09(16)
N(7)–Zn(1)–N(2)	172.8(2)
N(1)–Zn(1)–N(6)	96.3(2)
N(1)–Zn(1)–O(1)	87.8(2)

**Table 6:** Selected bond lengths (Å) and bond angles (°) of complex 2.

Bond	Dist.
Zn(1)–Br(1)	2.3834(8)
Zn(1)–Br(3)	2.3715(9)
Zn(1)–Br(2)	2.3878(9)
Zn(1)–N(1)	2.073(4)
S(1)–C(7)	1.726(5)
S(1)–C(8)	1.740(6)

Angle	(°)
Br(1)–Zn(1)–Br(2)	112.75(3)
Br(3)–Zn(1)–Br(1)	110.68(3)
Br(3)–Zn(1)–Br(2)	112.54(3)
N1–Zn(1)–Br(1)	109.01(12)
N1–Zn(1)–Br(3)	105.75(12)
N1–Zn(1)–Br(2)	105.70(12)

**Table 7:** Hydrogen bonds for the complex 2 (Å and °).

D–H···A	D–H	H···A	D···A	D–H···A
N(3)–H(3)···O(1)	0.8800	1.9800	2.793(16)	153.500
N(3)–H(3)···O(1A)	0.8800	1.9000	2.740(3)	160.400

**Table 8:** Bactericidal activity screening test levels of the ligands and complexes (100 *μ*g/mL)^a^.

Compounds	Inhibition ratio (%) (Levels^c^)						
	*E. coli* ^b^	*S. aureus* ^b^	*S. typhimurium* ^b^	*B. subtilis* ^b^	*Sh. flexneri* ^b^	*V. parahaemolyticus* ^b^	*P. aeruginosa* ^b^
**L1**	84.9	81.5	77.2	73.9	**96.3**	74.8	**97.1**
	(++)	(++)	(++)	(++)	(+++)	(++)	(+++)
**L2**	**101.7**	55.4	77.0	**99.1**	**98.7**	14.3	79.2
	(+)	(−)	(+)	(+++)	(+++)	(−)	(+)
**1**	73.9	**97.3**	**99.7**	**102.0**	**99.2**	**98.7**	43.8
	(++)	(+++)	(+++)	(+++)	(+++)	(+++)	(−)
**2**	**102.3**	**102.2**	**96.3**	54.6	**100.0**	40.4	**101.3**
	(+++)	(+++)	(+++)	(+++)	(+++)	(−)	(+++)

^a^Bactericidal activity is revealed by the percentage of complexes against bacterial strain. ^b^*Escherichia coli* (*E. coli*, ATCC 25922), *Staphylococcus aureus* (*S. aureus*, CMCC(B) 26003), *Salmonella typhimurium* (*S. typhimurium*, CMCC(B) 50071), *Bacillus subtilis* (*B. subtilis*, ATCC 6633), *Shigella flexneri* (*Sh. flexneri*, CMCC(B) 51572), *Vibrio parahaemolyticus* (*V. parahaemolyticus*, ATCC 17802), *Pseudomonas aeruginosa* (*P. aeruginosa*, ATCC 9027). ^c^Activity levels; +++ ≥ 90%; ++ ≥ 70–89%; + ≥ 50–69%; − < 50%.

**Table 9:** Tests of MIC (*μ*g/mL) of the ligands and complexes against bacterial strains^a^.

Compounds	MIC (*μ*g·mL^−1^)						
	*E. coli*	*S. aureus*	*S. typhimurium*	*B. subtilis*	*Sh. flexneri*	*V. parahaemolyticus*	*P. aeruginosa*
CIPRO^b^	12.5	6.25	12.5	6.25	3.13	3.13	6.25
**L1**	—	—	—	—	50.0	—	100.0
**L2**	12.5	—	—	50	100	—	—
**1**	—	12.5	6.25	3.13	12.5	12.5	—
**2**	3.13	12.5	12.5	—	3.13	—	6.25

^a^Results are expressed as the minimum inhibitory concentration (MIC). ^b^Ciprofloxacin (CIPRO).

**Table 10:** Tests of IC_50_ (*μ*g/mL) of the ligands and complexes against cancer cells^a^.

Compounds	IC_50_ ± SD (*μ*g·mL^−1^)			
	A549^b^	BT-20^b^	MCF-7^b^	U20S^b^
**L1**	30.16 ± 3.45	45.30 ± 4.84	26.50 ± 4.49	52.00 ± 4.37
**L2**	36.72 ± 2.36	23.25 ± 1.63	21.33 ± 3.81	38.22 ± 4.12
**1**	7.43 ± 1.55	12.23 ± 2.11	17.35 ± 3.23	9.78 ± 1.24
**2**	4.48 ± 0.68	8.23 ± 1.02	6.38 ± 0.29	9.52 ± 0.93

^a^Results are expressed as the half maximal inhibitory concentration (IC_50_). ^b^Human lung cancer cells (A549), human breast cancer cells (BT-20), human ovarian cancer cells (MCF-7), human osteosarcoma cells (U20S).

## Data Availability

The data used to support the findings of this study are available from the corresponding author upon request.

## Supplementary Materials

Supplementary Materials Supplementary data include MOL files and InChiKeys of the most important compounds described in this article. **CCDC: 2005334, L2; 1997625, 1; 2005335, 2** contain supplementary crystallographic data for **L2** and complexes **1-2**, respectively. These data can be obtained free of charge via or the Cambridge Crystallographic Data Centre, 12 Union Road, Cambridge CB2 1EZ, UK; fax: (+44) 1223-336-033; or e-mail through deposit@ccdc.cam.ac.uk.
