# Supplementary material for: Synthesis, Crystal Structures, and Antimicrobial and Antitumor Studies of Two Zinc(II) Complexes with Pyridine Thiazole Derivatives
Source: Bioinorg Chem Appl. 2020 Sep 15;2020:8852470. doi: 10.1155/2020/8852470 (PMC7512040; doi:10.1155/2020/8852470)
Supplement: Supplementary Materials — Supplementary data include MOL files and InChiKeys of the most important compounds described in this article. CCDC: 2005334, L2; 1997625, 1; 2005335, 2 contain supplementary crystallographic data for L2 and complexes 1-2, respectively. These data can be obtained free of charge via or the Cambridge Crystallographic Data Centre, 12 Union Road, Cambridge CB2 1EZ, UK; fax: (+44) 1223-336-033; or e-mail through deposit@ccdc.cam.ac.uk. [file 8852470.f1.doc]

**Supplementary Information (SI)**


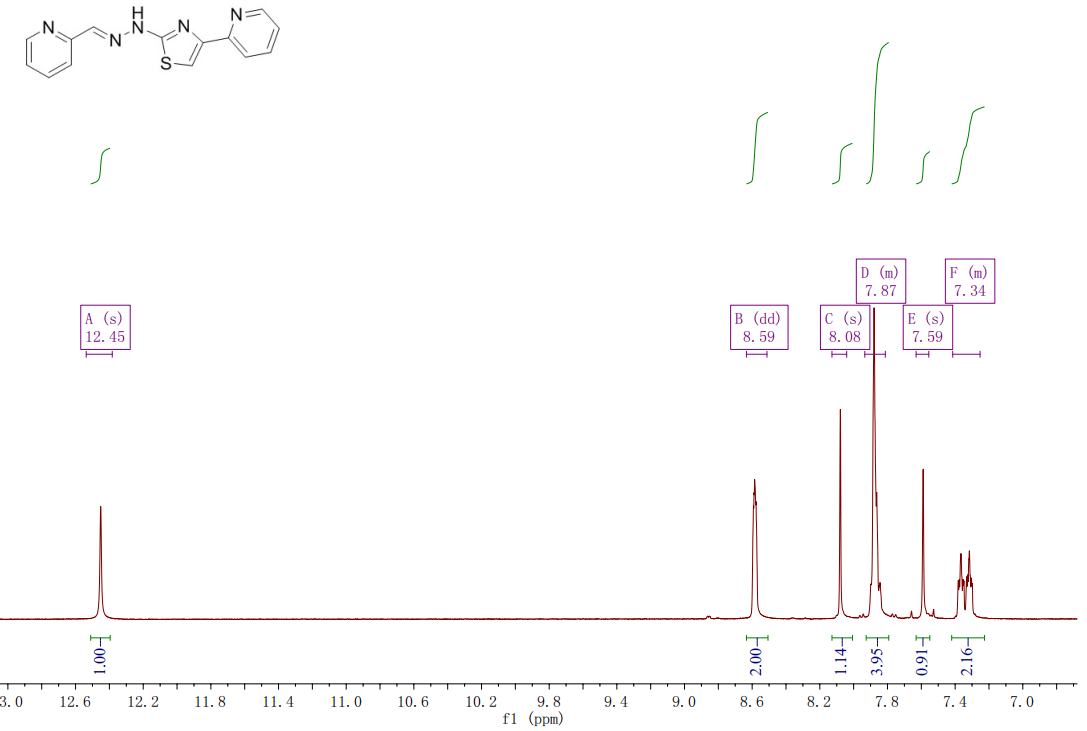


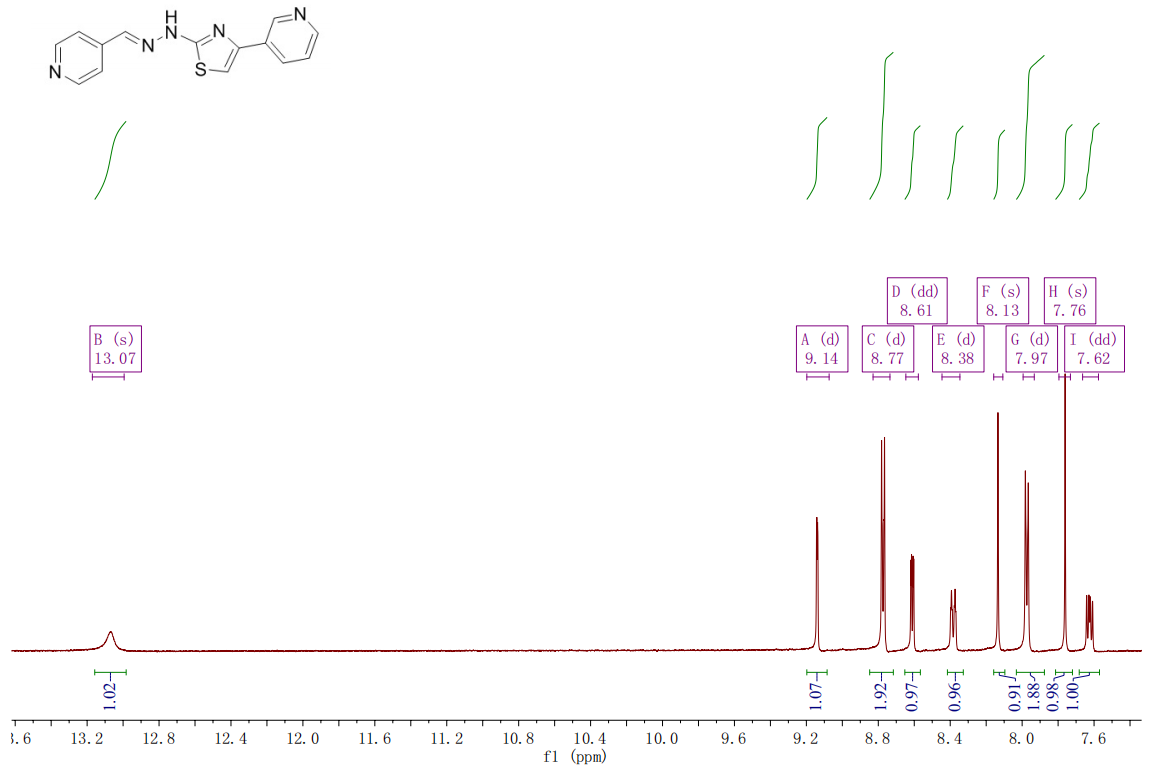


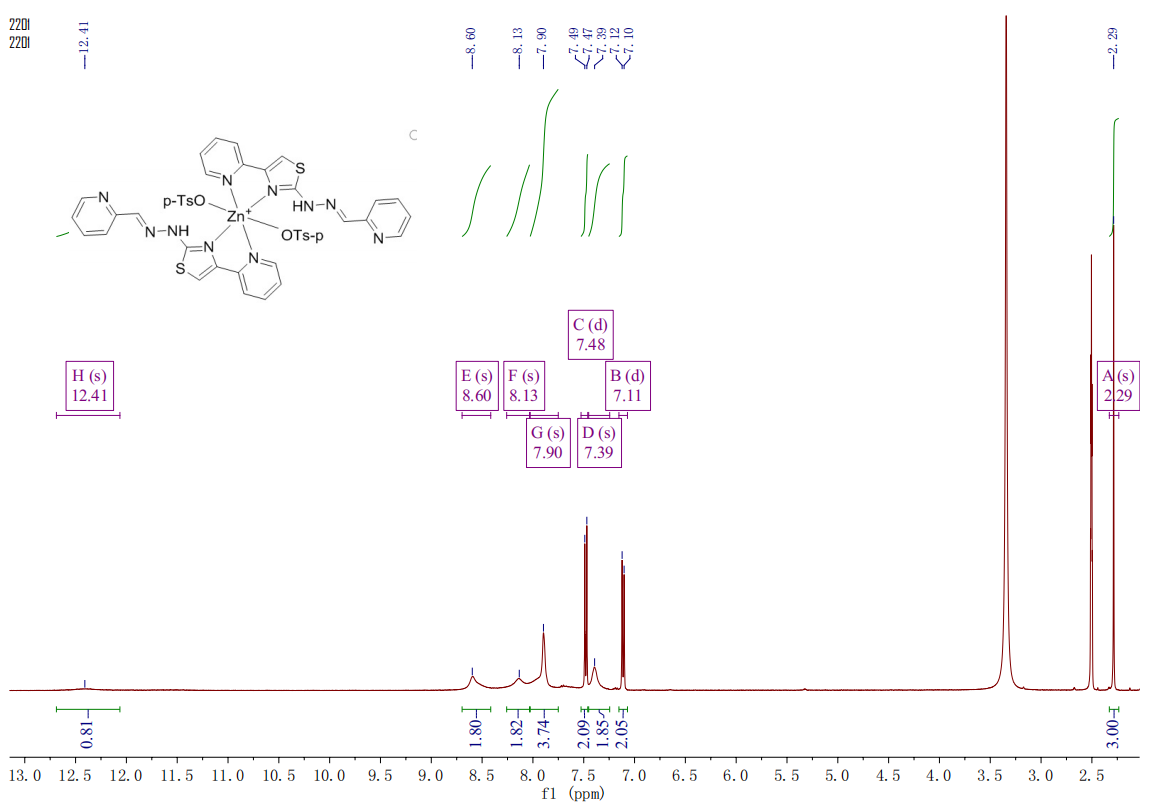


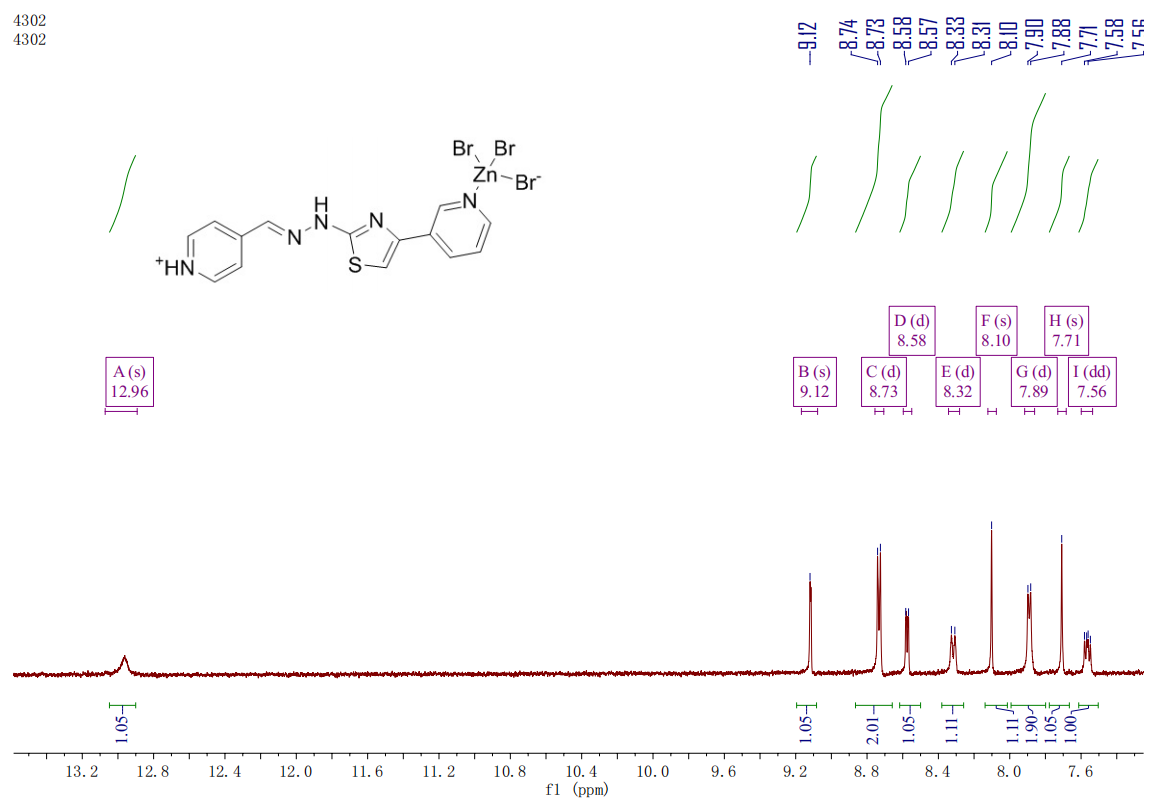


**Fig. S1 The ^1^H-NMR spectrum of the ligand L1-L2 and complexes 1-2.**


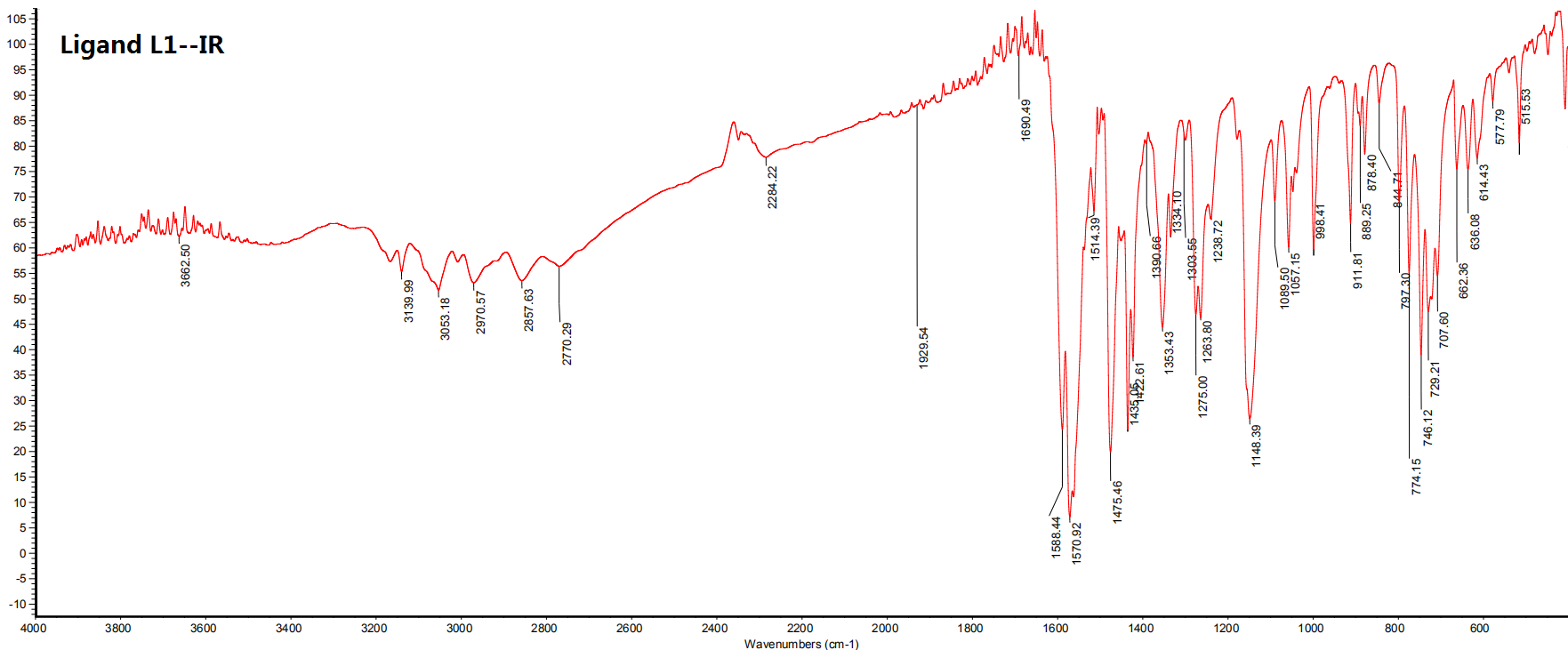

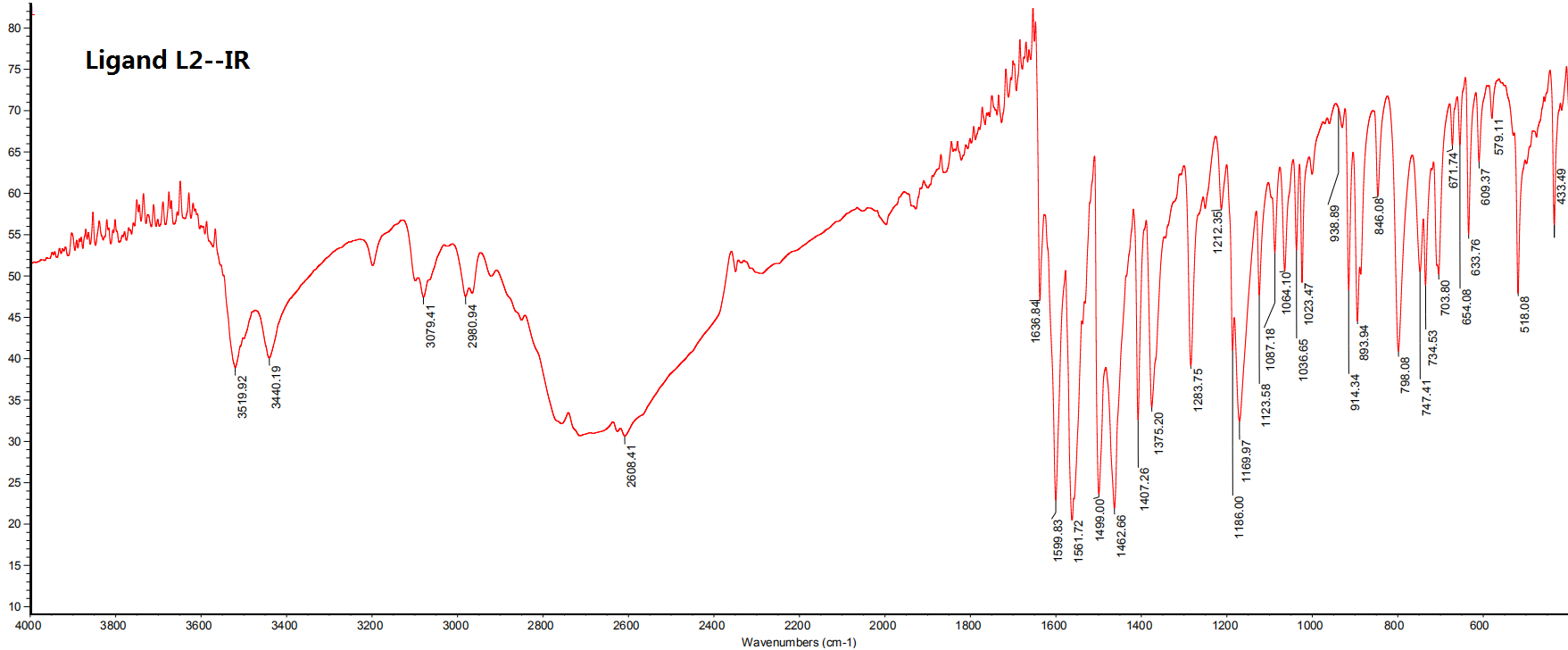

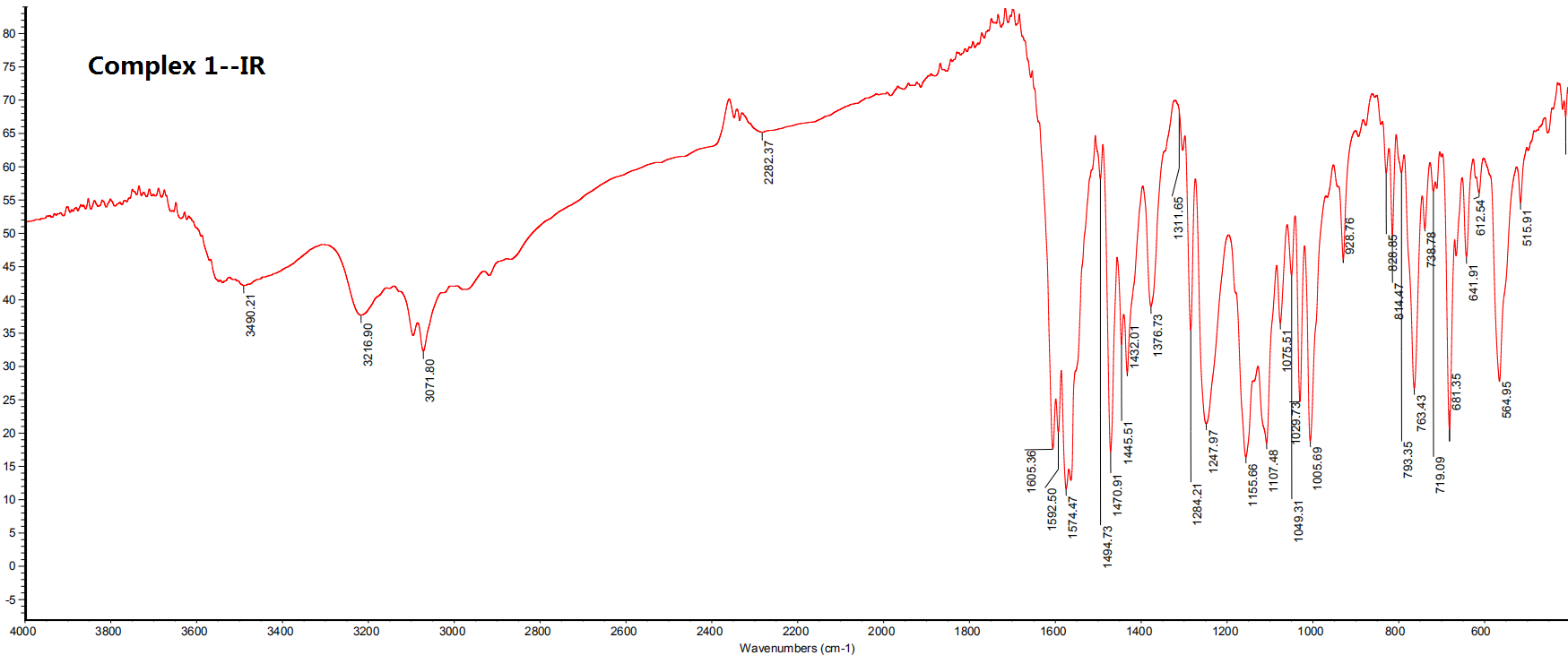

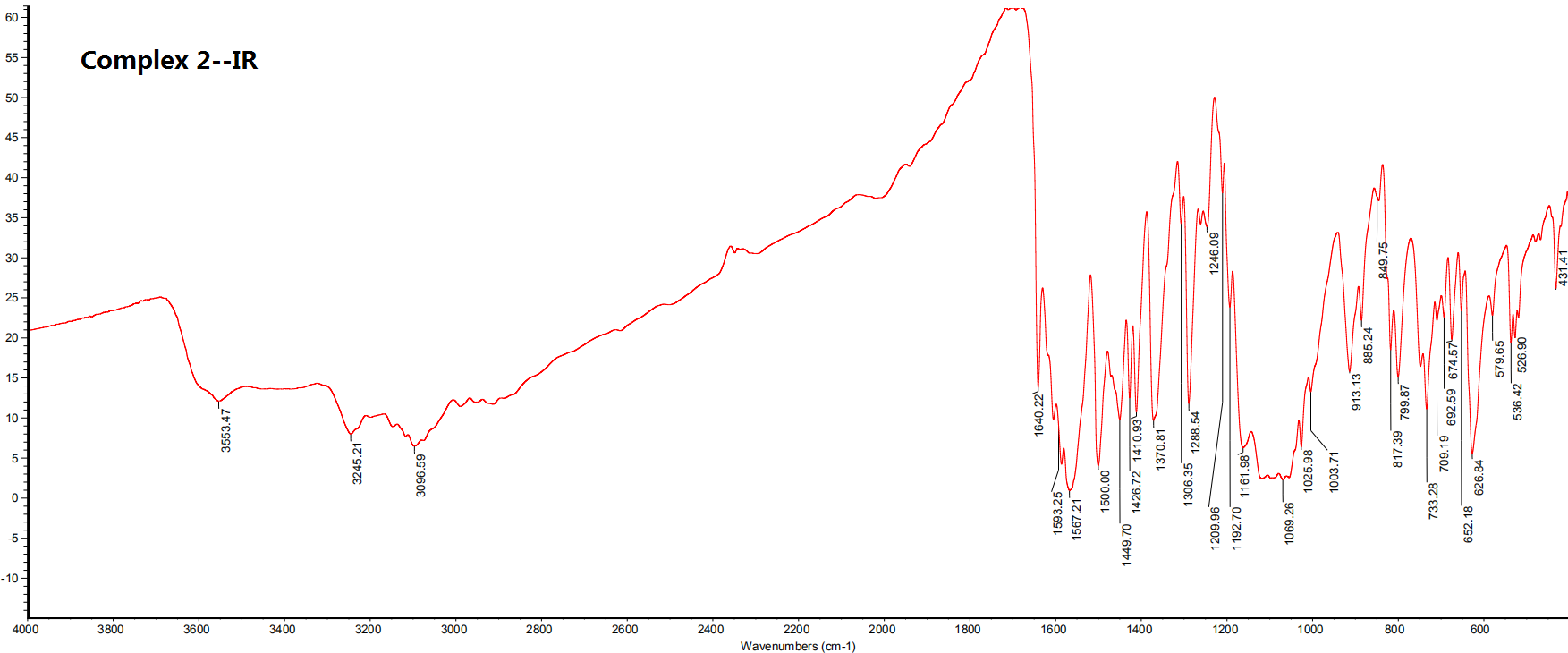


**Fig. S2 The FTIR spectrum of the ligand L1-L2 and complexes 1-2.**

**
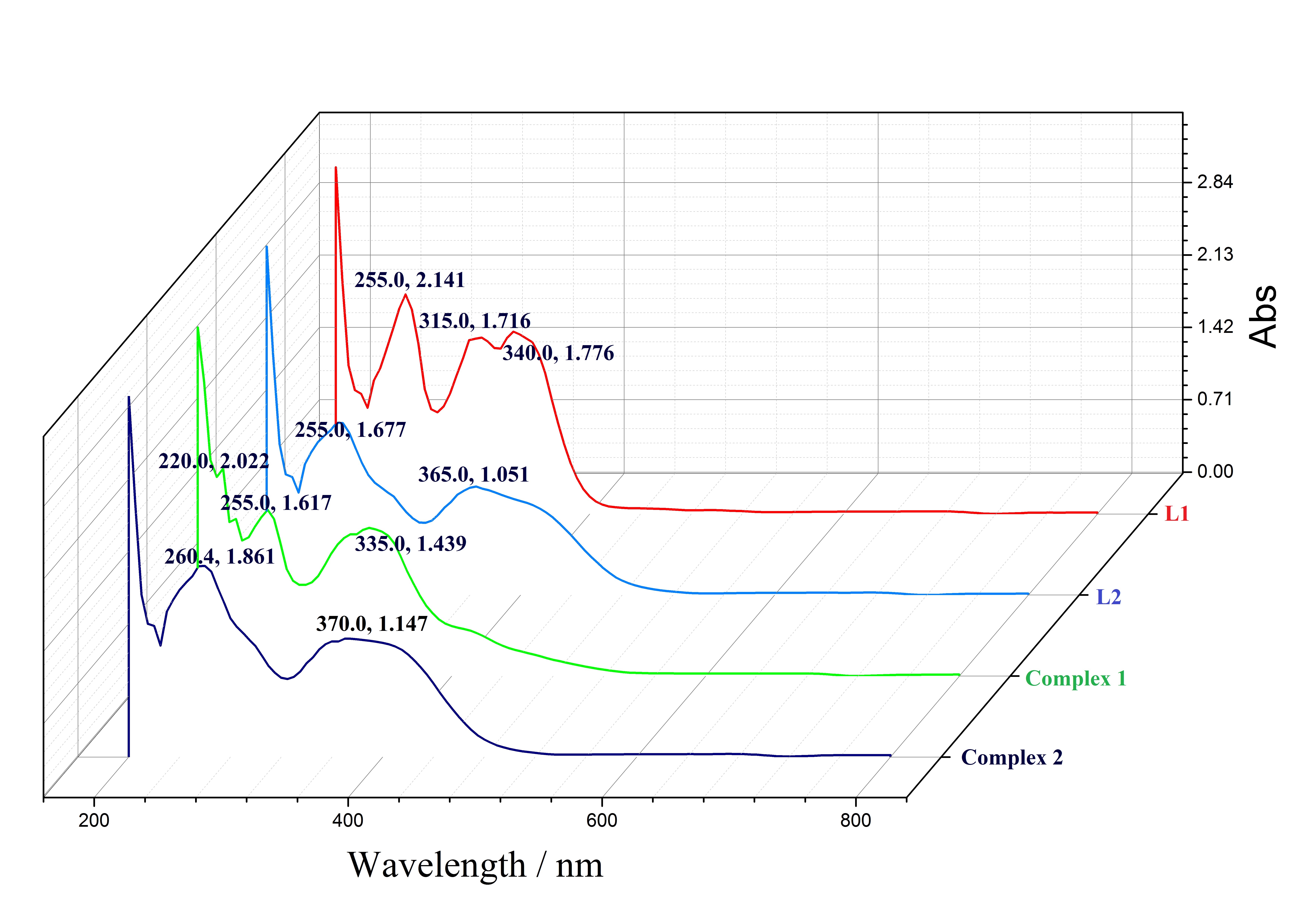
**

**Fig. S3 The UV-Vis absorption** **spectrum of the ligand L1-L2 and complexes 1-2.**

**
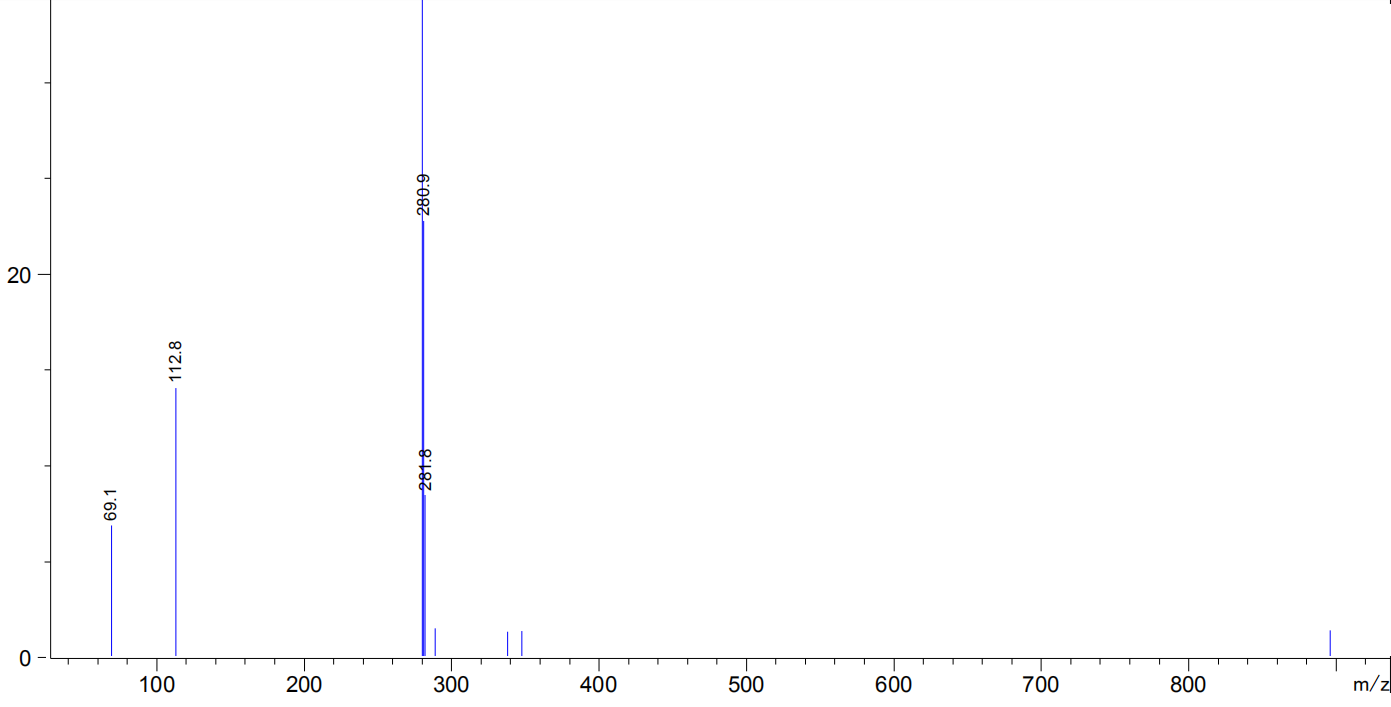

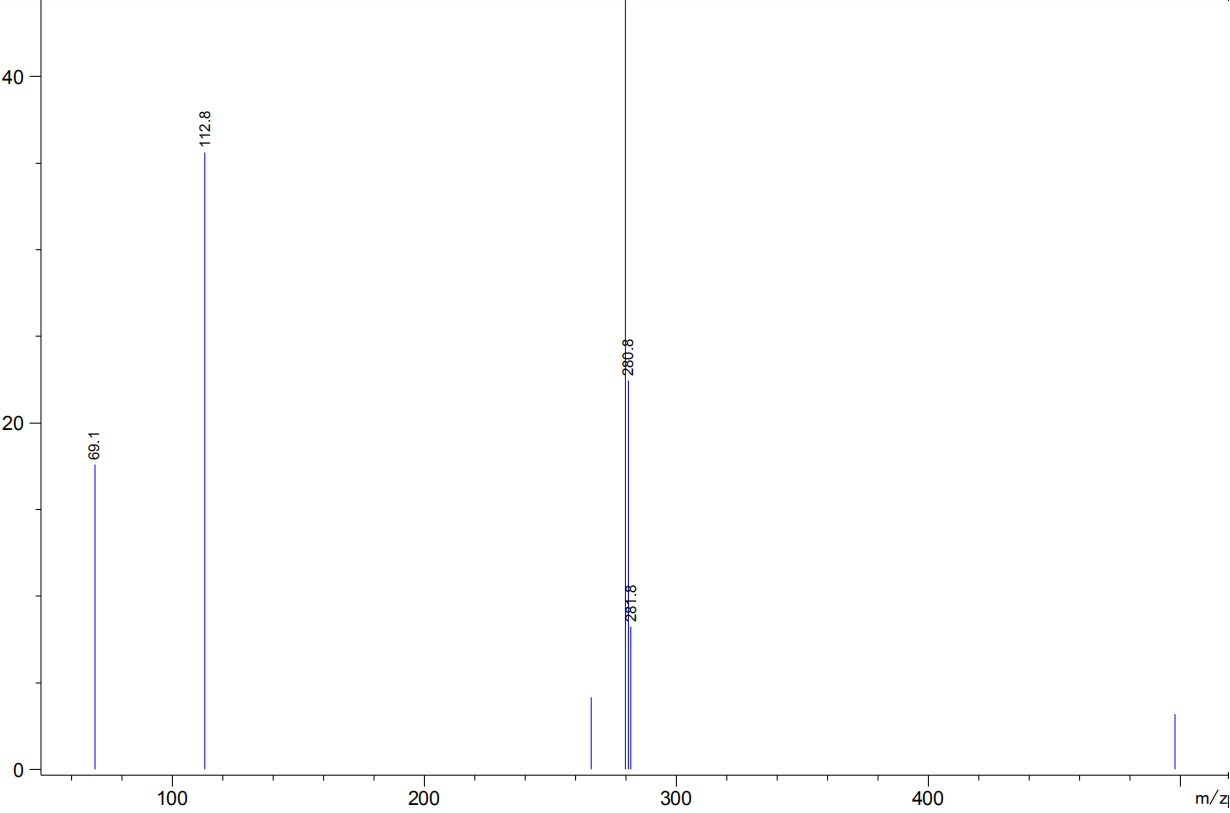
**

**Fig. S4 The MS spectrum of the ligand L1-L2**
